# The effect of family integrated care based on Swanson theory of caring on premature infants: A non-randomized cohort study

**DOI:** 10.1097/MD.0000000000049889

**Published:** 2026-07-24

**Authors:** Lili Chen, Huijie Zhu

**Affiliations:** a Yongkang Women and Children’s Health Hospital, Yongkang, Zhejiang, China.

**Keywords:** cohort study, family integrated care, premature infants, repeated measures ANOVA

## Abstract

Preterm birth significantly impacts neonatal growth and development, and the family integrated care (FICare) nursing model offers several benefits. This study investigated the impact of FICare on preterm infants, based on Swanson caring theory. This theory includes 5 core processes (knowing, being with, doing for, enabling, and maintaining belief) that guide person-centered care. A non-randomized cohort study was conducted. A total of 52 preterm infants (28–36 weeks of gestation) were enrolled, 33 in the intervention group (FICare nursing model based on Swanson caring theory) and 19 in the control group (traditional care). The primary outcomes were the length, weight, and head circumference of preterm infants at corrected ages of 1, 3, and 6 months. Additionally, parental anxiety levels and the Parenting Sense of Competence scale were assessed. The intervention group demonstrated a shorter transitional feeding period (*P* = .015), a higher breastfeeding rate (*P* = .007), and a significantly lower rate of unplanned rehospitalization (*P* = .004). Infants in the intervention group had significantly greater body length (*P* = .041) and weight (*P* = .002) at corrected ages. Parental state and trait anxiety decreased, and Parenting Sense of Competence scores were increased (*P* < .001). No significant differences were observed in the duration of oxygen therapy, total enteral feeding period, length of hospital stay, or weight at discharge from hospital. FICare based on Swanson caring theory was associated with improved feeding transition, reduces unplanned readmissions, promotes growth within 6 months corrected age, alleviates parental anxiety, and enhances parenting competence. The findings are limited by the non-randomized design and baseline imbalance in birth weight.

## 1. Introduction

Preterm birth is defined as the birth of a live newborn before the completion of 37 weeks of gestation.^[1]^ Common causes of preterm birth include infections or inflammatory conditions, uteroplacental ischemia or hemorrhage, excessive uterine distension, maternal stress, and other immune-related mechanisms.^[2]^ Globally, there are approximately 15 million premature infants born each year, representing more than 10% of all newborns, and this number continues to rise.^[3]^ Preterm infants require specialized care in the neonatal intensive care unit (NICU), which often involves prolonged hospitalization and parent-infant separation. Research indicates that this situation can increase anxiety in both parents and newborns.^[4,5]^

Family integrated care (FICare) is designed to enhance the quality of care for premature infants by actively involving parents in clinical decision-making and providing them with parenting education and psychological support. The core principle of FICare is to empower parents to become the primary caregivers of their infants, enabling their full participation throughout the infants’ birth and hospitalization process.^[6]^ Swanson caring theory, developed by Dr Kristen M. Swanson, highlights the importance of genuine human connection in the nurse–patient relationship.^[7,8]^ Notably, it provides a structured, person-centered framework that addresses the emotional and relational needs of parents that standard FICare often overlooks. By emphasizing knowing, being with, and enabling, the intervention reduces parental anxiety,^[9]^ strengthens parent-infant attachment, and promotes consistent, responsive caregiving. These 5 caring processes transform FICare from simple parental task participation into relational, empathic, and empowering care, which may further improve infant growth and parental well-being.

Previous studies have demonstrated that the implementation of FICare in single-family neonatal units is associated with a reduced risk of late-onset sepsis among preterm infants,^[10]^ a shorter hospital length of stay,^[11]^ a lower risk of rehospitalization, and improved breastfeeding rates.^[12]^ However, most previous FICare studies focused on in-hospital outcomes such as sepsis, parental psychological outcomes, and breastfeeding rate. Few studies have explored long-term growth up to 6 months corrected age with FICare guided by Swanson caring theory. To address this gap, a cohort study was conducted.

## 2. Methods

### 2.1. Population

This was a non-randomized cohort study conducted in a real-world clinical setting. Randomization was not performed due to parental acceptance of the intervention. Group allocation was based on parental willingness to participate in Swanson theory-guided FICare program: infants whose parents agreed to participate were assigned to the intervention group, and those who declined were assigned to the control group. Written informed consent was obtained from the parents of all participating preterm infants. The inclusion criteria were as follows: gestational age ranging from 28 to 36 weeks, stable vital signs for at least 24 hours, and parental accompaniment of preterm infants during hospitalization. The exclusion criteria included: preterm infants with severe congenital or genetic disorders, dysfunction of vital organs, or requiring mechanical life support; preterm infants requiring surgical intervention; and preterm infants with a birth weight below 500 g. Enrollment was suspended once the target sample sizes were achieved in both groups.

### 2.2. Intervention group

The FICare intervention was strictly mapped to the 5 core processes of Swanson caring theory (knowing, being with, doing for, enabling, and maintaining belief). To ensure standardized implementation and replicability, the nursing supervision team developed a dedicated intervention fidelity checklist. The head nurse carried out checks and on-site evaluations on a weekly basis. Detailed and standardized operational procedures were formulated to ensure clinical implementation:

1. Knowing: A designated neonatologist offered a detailed explanation to parents within 24 hours after birth, covering the infant’s gestational age, physical condition, potential complications, feeding requirements, and developmental prognosis.
2. Being with: Two specialized NICU nurses offered daily bedside accompaniment to parents for 30 minutes per session, addressed their emotional needs, alleviated anxiety and helplessness, and established a trustworthy medical-family relationship.
3. Doing for: A neonatal nurse provided step-by-step guidance for parents to participate in daily care, including oral feeding, skin care, posture management, and diaper care. Guidance was provided once daily until parents mastered the skills.
4. Enabling: A full-time psychological counselor provided individualized psychological counseling (twice weekly) for parents, offered emotional support and stress relief guidance, and helped parents access social support and professional parenting resources.
5. Maintaining belief: Neonatologists and nurses conducted outpatient follow-up and telephone follow-up (at 1, 3, and 6 months of corrected age) to dynamically monitor the growth of infants, confirm family care behaviors, and sustain parental confidence in caregiving.

### 2.3. Comparison group

The control group received standard neonatal intensive care without Swanson theory guidance or structured family integrated care. The specific measures were as follows:

1. Monitoring: Vital signs, blood oxygen, feeding tolerance, and weight were monitored by NICU nurses according to hospital standards.
2. Nutritional support: Routine enteral feeding and oxygen therapy were provided based on the infant’s condition.
3. Parental education: Brief education was provided to parents before discharge, including basic feeding and home care tips.

In comparison with the intervention group, the comparison group did not receive fixed accompanying support, specialized psychological counseling, systematic parenting skills training, or extended post-discharge follow-up support.

### 2.4. Outcome

Primary outcome: The head circumference, body weight, and body length of the premature infants were assessed at adjusted ages of 1, 3, and 6 months (corrected gestational age for prematurity). The Trait Anxiety Inventory (T-AI), which assesses long-term and stable anxiety, and the State Anxiety Inventory (S-AI), which evaluates current or recent anxiety levels, were employed. Additionally, the Parenting Sense of Competence (PSOC) scale was utilized to measure parental self-efficacy and satisfaction with their parenting role.

Secondary outcome: Length of hospital stay, discharge weight, unplanned hospital readmission, duration of oxygen therapy, time to achieve full enteral feeding, and transition time to competent oral feeding.

Confounding factors: Gender, gestational age, and birth weight of premature infants.

### 2.5. Sample size calculation

The sample size calculation was performed with the assistance of Select Statistical Services (https://select-statistics.co.uk/calculators/sample-size-calculator-two-means/), based on the primary outcome: body weight of preterm infants at the corrected age of 6 months. The formula is:


$$n=\frac{2\times {\left(Z_{\alpha /2}+Z_{\beta }\right)}^{2}\times \sigma^{2}}{d^{2}}$$

Where “*Z*_α/2_” is the critical value of the normal distribution at α/2 (for a confidence level of 95%, α is 0.05 and the critical value is 1.96); “*Z*_β_” is the critical value of the normal distribution at β (for a power of 80%, β is 0.2 and the critical value is 0.84), “σ^2^” is the population variance (is 2.5), and “*d*” is the difference you would like to detect (set to 1.5 in primary outcome). A minimum sample size of 18 participants per group was calculated.

### 2.6. Blinding

Since parents, neonatologists, neonatal nurses, and psychologists participated in the entire intervention process, they were not blinded. However, the follow-up doctors and nurses, who measured and collected the growth indicators of the newborns and evaluated the anxiety levels of the parents, were unaware of the study groups.

### 2.7. Statistical analysis

Measurement data that conformed to a normal distribution were expressed as mean ± standard deviation. Independent and paired sample *t* tests were conducted to assess statistical differences between groups. For measurement data that did not conform to a normal distribution, results were presented as median (interquartile range), and the Mann–Whitney *U* test was used to evaluate statistical differences. Categorical count data were summarized as n (%) and compared using the chi-square test. Repeated measurement data were analyzed using repeated measures analysis of variance (ANOVA). Linear regression analysis was employed to assess the independent factors influencing the neonatal developmental index of premature infants, as well as the parental anxiety scores and sense of parenting competence. All multivariable models included the intervention variable and all baseline imbalanced variables to adjust for confounding caused by nonrandom group allocation. Statistical analyses were performed using SPSS 25.0 software (IBM Corporation). Post hoc power analyses for the key comparisons were estimated using the pwr package in R (version 4.5.3; The R Foundation for Statistical Computing).

## 3. Results

A total of 52 premature infants were enrolled in the study. Among them, 33 infants received the FICare nursing approach guided by Swanson theory, while 19 infants received traditional nursing care. All 52 enrolled preterm infants completed all scheduled follow-up visits at corrected ages of 1, 3, and 6 months. No participants were lost to follow-up during the whole study period, and all growth measurements were fully completed with no missing data.

No significant differences were observed between the 2 groups in terms of gender or gestational age. During the nursing period, no significant differences were found in the duration of oxygen therapy, full enteral feeding time, length of hospital stay, or discharge weight between the 2 groups. Baseline analysis showed significant between-group differences in birth weight (1.55 ± 0.41 vs 1.79 ± 0.32, *P* = .03), transition time to competent oral feeding (10.7 ± 5.3 vs 14.8 ± 6.4, *P* = .015), unplanned readmission rate (3.0% vs 31.6%, *P* = .004), and feeding patterns (*P* = .007), as shown in Table 1. Follow-up data revealed that infants who received Swanson theory-guided nursing exhibited significantly greater head circumference, weight, and length at the same adjusted age compared to the control group, as presented in Table 1.

**Table 1:** Baseline, preterm infant development and parental anxiety status between the 2 groups.

	Intervention (n = 33)	Control (n = 19)	*P* value	Power
Gender (male)	15 (45.5%)	11 (57.9%)	.388	
Gestational age	219.5 ± 16.6	227.8 ± 12.4	.064	
Birth weight	1.55 ± 0.41	1.79 ± 0.32	.03	
Duration of oxygen therapy	30 (42.5)	11 (32)	.066	
Full enteral feeding time	10.0 ± 5.0	8.26 ± 4.0	.210	
Transition time to competent oral feeding	10.7 ± 5.3	14.8 ± 6.4	.015	
Discharge weight	2.98 ± 0.49	2.83 ± 0.54	.323	
Length of hospital stay	41 (37.5)	31 (18)	.143	
Unplanned readmissions	1 (3.0%)	6 (31.6%)	.004	
Feeding patterns			.007	
Exclusive breastfeeding	19 (57.6%)	3 (15.8%)		
Mixed breast milk	3 (9.1%)	2 (10.5%)		
Non-breastfeeding	11 (33.3%)	14 (73.7)		
Adjusted head circumference				
1 mo	37.8 ± 0.88	36.4 ± 0.99	<.001*	1.0
3 mo	41.1 ± 0.81	39.3 ± 1.02	<.001*	1.0
6 mo	43.6 ± 1.12	42.0 ± 0.88	<.001*	1.0
Adjusted body length				
1 mo	55.6 ± 2.53	53.6 ± 1.65	.003*	0.86
3 mo	64.8 ± 2.35	61.1 ± 1.59	<.001*	1.0
6 mo	69.3 ± 2.63	67.5 ± 2.07	.036*	0.71
Adjusted body weight				
1 mo	4.87 ± 0.41	4.26 ± 0.40	<.001*	1.0
3 mo	6.65 ± 0.70	6.06 ± 0.31	<.001*	0.93
6 mo	8.59 ± 0.53	7.47 ± 0.42	<.001*	1.0
PSOC scores of mother				
1 day following delivery	69.7 ± 4.8	70.7 ± 4.7	.884*	
Date of discharge	89.6 ± 4.2	71.3 ± 4.7	<.001*	1.0
PSOC scores of father				
1 day following delivery	71.0 ± 6.4	71.5 ± 6.9	1.0*	
Date of discharge	90.0 ± 5.4	72.3 ± 9.8	<.001*	1.0
S-AI of mother				
1 day following delivery	58.8 ± 5.1	53.6 ± 5.1	.002*	
Date of discharge	41.4 ± 4.1	52.5 ± 5.2	<.001*	1.0
T-AI of mother				
1 day following delivery	49.4 ± 6.1	48.3 ± 6.0	1.0*	
Date of discharge	37.2 ± 4.8	47.5 ± 5.7	<.001*	1.0
S-AI of father				
1 day following delivery	54.3 ± 5.8	52.3 ± 10.2	.847*	
Date of discharge	40.6 ± 4.6	54.3 ± 8.8	<.001*	1.0
T-AI of father				
1 day following delivery	43.6 ± 5.0	41.3 ± 4.5	.220*	
Date of discharge	39.1 ± 5.1	41.8 ± 5.4	.154*	0.42

PSOC = Parenting Sense of Competence scale, S-AI = State Anxiety Inventory, T-AI = Trait Anxiety Inventory.

* Adjusted by Bonferroni methods.

Additionally, the parental questionnaire survey revealed that there was no significant difference in anxiety levels or parenting sense of competence scores between the 2 groups on the 1st day after delivery. After nursing interventions during hospitalization, significant differences emerged between the 2 groups in both anxiety levels and parenting sense of competence, as presented in Table 1. Furthermore, comparative analysis before and after the nursing intervention indicated that the nursing approach guided by Swanson theory effectively reduced parental anxiety scores and enhanced the parenting sense of competence. In the control group, no significant changes were observed in the fathers’ T-AI, mothers’ T-AI, or PSOC scores; however, an increase was noted in the fathers’ S-AI scores. Detailed results are provided in Table 2.

**Table 2:** Changement of PSOC and STAI scores before and after nursing intervention.

	One day following delivery	Date of discharge	*P* value
Intervention group			
PSOC scores of mother	69.7 ± 4.8	89.6 ± 71.3	<.001
PSOC scores of father	71.0 ± 6.4	90.0 ± 5.4	<.001
S-AI scores of mother	58.8 ± 5.1	41.4 ± 4.1	<.001
T-AI scores of mother	49.4 ± 6.1	37.2 ± 4.8	<.001
S-AI scores of father	54.3 ± 5.8	40.6 ± 4.6	<.001
T-AI scores of father	43.6 ± 5.0	39.1 ± 5.1	<.001
Control group			
PSOC scores of mother	70.7 ± 4.7	71.3 ± 4.7	.609
PSOC scores of father	71.5 ± 6.9	72.3 ± 9.8	.276
S-AI scores of mother	53.6 ± 5.1	52.5 ± 5.2	.226
T-AI scores of mother	48.3 ± 6.0	47.5 ± 5.7	.225
S-AI scores of father	52.3 ± 10.2	54.3 ± 8.8	<.001
T-AI scores of father	41.3 ± 4.5	41.8 ± 5.4	.540

PSOC = Parenting Sense of Competence scale, S-AI = State Anxiety Inventory, T-AI = Trait Anxiety Inventory.

**Figure 1.**
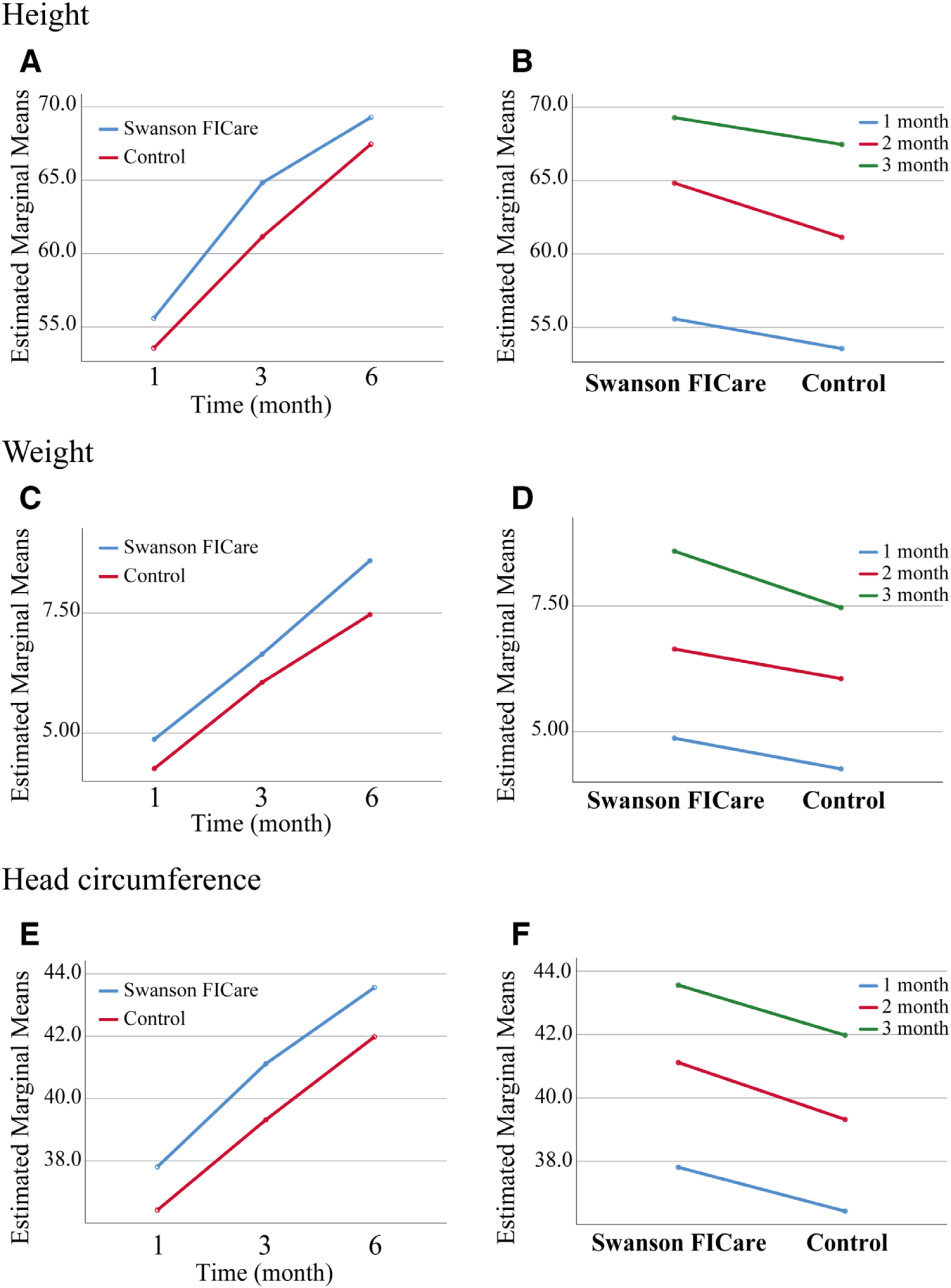
Estimated marginal means from repeated measures ANOVA. (A and B) Line plots showing the estimated marginal mean effects of preterm body length at varying corrected ages. (C and D) Line plots depicting the estimated marginal mean effects of preterm birth weight across different corrected ages. (E and F) Line plots illustrating the estimated marginal mean effects of head circumference in 2 groups of preterm infants at various corrected age points. ANOVA = analysis of variance, FICare = family integrated care.

The simple independent sample *t* test and paired sample *t* test conducted before and after the intervention were insufficient to accurately reflect the effects of time and the intervention, as both time and exposure had significant influences on head circumference, weight, and length. Therefore, repeated measures ANOVA was employed to evaluate the effects of time and the FICare nursing intervention based on Swanson theory of caring. Mauchly test of sphericity indicated that the assumption of sphericity was met for the repeated measures of head circumference, body length, and weight; thus, the within-subject effects were assessed using the sphericity-assumed estimates. The analysis revealed a significant time effect, indicating that head circumference, body length, and weight increased progressively over time, which aligns with clinical expectations. Additionally, significant between-group differences were observed in the body length (*P* = .041) and weight (*P* = .002) of preterm infants. As presented in Table 3, statistically significant differences in body length and weight were found at each time point between the 2 groups, suggesting that the FICare nursing approach guided by Swanson theory positively contributes to the growth and development of preterm infants. The estimated marginal means from the repeated measures ANOVA are illustrated in Figure 1.

**Table 3:** Repeated measures ANOVA of growth and developmental indicators in preterm infants.

	1month (n = 52)	3 mo (n = 52)	6 mo (n = 52)	*P*_Mauchly_	*P*_time_	*P*_group*time_
Head circumference				.200	<.001	.462
Intervention	37.8 ± 0.88	41.1 ± 0.81	43.6 ± 1.12			
Control	36.4 ± 0.99	39.3 ± 1.03	42.0 ± 0.88			
*P*_time point_	<.001*	<.001*	<.001*			
Body length				.135	<.001	.041
Intervention	55.6 ± 2.53	64.8 ± 2.35	69.3 ± 2.63			
Control	53.6 ± 1.65	61.1 ± 1.59	67.5 ± 2.07			
*P*_time point_	.003*	<.001*	.036*			
Body weight				.493	<.001	.002
Intervention	4.87 ± 0.41	6.65 ± 0.70	8.59 ± 0.53			
Control	4.26 ± 0.40	6.06 ± 0.31	7.47 ± 0.42			
*P*_time point_	<.001*	.001*	<.001*			

ANOVA = analysis of variance.

* Adjusted by Bonferroni methods.

Table 1 indicates that there is a difference in birth weight between the FICare nursing group guided by Swanson theory and the control group of premature infants. Furthermore, the FICare nursing method based on Swanson theory may influence neonatal transitional feeding duration, unplanned readmission rates, and feeding patterns, which in turn could affect the infants’ subsequent growth and development. These variables were considered as potential mediators in the repeated measures ANOVA analysis. For the primary growth outcomes (weight at corrected 6 months), the effect size was large (Cohen *d* = 1.82), and the achieved power exceeded 0.99, confirming the robustness of the significant findings. The statistical power of other indicators was also evaluated.

Therefore, linear regression models were employed to assess the impact of these factors on neonatal growth and parental anxiety. As shown in Table 4, after adjusting for all baseline imbalanced variables, Swanson theory-guided FICare nursing approach was identified as an independent factor influencing head circumference, body length, and weight gain in infants at corrected ages of 1, 3, and 6 months. Additionally, this intervention was associated with reduced parental anxiety and enhanced parental sense of competence. A significant correlation was observed between neonatal birth weight and head circumference, length, and weight at the adjusted age of 1 month, as well as length and weight at 3 months; however, this association was no longer statistically significant at 6 months. Shorter transitional feeding duration was associated with greater head circumference and weight development at 1 month adjusted age, but this relationship lost statistical significance by 3 months. Unplanned readmission was found to be positively correlated with maternal anxiety levels.

**Table 4:** Linear regression model of outcome-related factors.

	Intervention	Birth weight	Transition time to competent oral feeding	Unplanned readmissions	Feeding patterns
	*P* value, *B* value (95% CI)	*P* value, *B* value (95% CI)	*P* value, *B* value (95% CI)	*P* value, *B* value (95% CI)	*P* value, *B* value (95% CI)
Adjusted head circumference					
1 mo (n = 52)	<.001, 1.843 (1.209–2.477)	.004, 0.990 (0.334–1.645)	.018, 0.054 (0.010–0.098)	.456, −0.288 (−1.056 to 0.481)	.518, 0.090 (−0.188 to 0.368)
3 mo (n = 52)	<.001, 1.748 (1.069–2.427)	.183, 0.472 (−0.231 to 1.174)	.426, −0.019 (−0.066 to 0.028)	.602, −0.215 (−1.038 to 0.609)	.848, −0.029 (−0.326 to 0.269)
6 mo (n = 52)	<.001, 1.858 (1.063–2.653)	.211, 0.158 (−0.305 to 1.341)	.988, 0.001 (−0.055 to 0.056)	.918, 0.050 (−0.915 to 1.014)	.350, 0.164 (−0.185 to 0.513)
Adjusted body length					
1 mo (n = 52)	.001, 2.828 (1.225–4.431)	.017, 2.038 (0.379–3.696)	.131, 0.086 (−0.026 to 0.197)	.228, −1.179 (−3.122 to 0.765)	.329, 1.669 (−0.359 to 3.696)
3 mo (n = 52)	<.001, 3.710 (2.162–5.258)	.036, 1.722 (0.120–3.324)	.861, −0.009 (−0.118 to 0.099)	.189, −1.244 (−3.121 to 0.633)	.995, −0.003 (−0.682 to 0.677)
6 mo (n = 52)	.038, 1.787 (0.105–3.468)	.458, −0.646 (−2.386 to 1.094)	.351, 0.055 (−0.062 to 0.172)	.018, −2.482 (−4.520 to −0.443)	.054, 0.724 (−0.014 to 1.462)
Adjusted body weight					
1 mo (n = 52)	<.001, 0.871 (0.594–1.147)	.016, 0.355 (0.069–0.641)	.005, 0.028 (0.009–0.047)	.933, −0.014 (−0.349 to 0.321)	.217, 0.076 (−0.046 to 0.197)
3 mo (n = 52)	.006, 0.609 (0.181–1.036)	.013, 0.566 (0.124–1.008)	.822, −0.004 (−0.033 to 0.026)	.210, −0.327 (−0.845 to 0.191)	.905, −0.012 (−0.199 to 0.176)
6 mo (n = 52)	<.001, 1.224 (0.866–1.582)	.192, 0.244 (−0.127 to 0.615)	.216, 0.016 (−0.009 to 0.041)	.136, −0.328 (−0.762 to 0.107)	.278, 0.086 (−0.071 to 0.243)
PSOC score at the time of discharge					
Mother	<.001, 19.916 (16.725–23.106)	.903, 0.201 (−3.100 to, 3.502)	.594, −0.060 (−0.282 to 0.163)	.021, 4.599 (0.731–8.467)	.397, 0.596 (−0.805 to 1.996)
Father	<.001, 17.737 (12.432–23.041)	.130, −4.207 (−9.695 to 1.282)	.121, −0.291 (−0.661 to 0.079)	.341, 3.077 (−3.355 to 9.508)	.145, 1.717 (−0.612 to 4.045)
S-AI score at discharge					
Mother	<.001, −9.323 (−12.410 to −6.235)	.576, 0.895 (−2.299 to 4.089)	.066, −0.202 (−0.418 to 0.014)	.018, 4.544 (0.801–8.287)	.062, 1.285 (−0.070 to 2.640)
Father	<.001, −13.567 (−18.446 to −8.687)	.850, −0.477 (−5.525 to 4.572)	.185, −0.228 (−0.568 to 0.113)	.465, 2.168 (−3.748 to 8.083)	.540, 0.657 (−1.485 to 2.798)
T-AI score at discharge					
Mother	<.001, −9.802 (−13.771 to −5.832)	.813, 0.485 (−3.622 to 4.592)	.932, 0.012 (−0.265 to 0.289)	.223, 2.956 (−1.857 to 7.769)	.461, −0.644 (−2.386 to 1.098)
Father	.123, −3.005 (−6.850 to 0.841)	.062, −3.785 (−7.764 to 0.194)	.674, −0.057 (−0.325 to 0.212)	.084, 4.088 (−0.574 to 8.750)	.678, −0.351 (−2.038 to 1.337)

CI = confidence intervals, PSOC = Parenting Sense of Competence scale, S-AI = State Anxiety Inventory, T-AI = Trait Anxiety Inventory..

## 4. Discussion

Parent-child separation represents a significant source of psychological stress for parents of premature infants and exerts adverse effects on the health and development of neonates.^[13,14]^ These effects may persist into adulthood, manifesting as increased susceptibility to psychological disorders^[15]^ and irritable bowel syndrome.^[16]^ Grounded in the principles of family-centered care, the FICare model fosters parental involvement through education, shared decision-making, and enhanced parenting experiences. Research indicates that FICare contributes to improved parental self-efficacy and stronger parent–infant relationships at hospital discharge. Guided by Swanson caring nursing theory, we integrated authentic interpersonal engagement with FICare to develop a comprehensive nursing approach centered on the 5 fundamental caring processes: maintaining belief, knowing, being with, doing for, and enabling. This study aimed to evaluate the impact of this integrated nursing method on the growth and development of preterm infants, parental anxiety levels, and parental sense of competence.

Our findings demonstrated that FICare, guided by Swanson caring theory, significantly reduced the transition time to competent oral feeding, lowered the risk of unplanned admissions, and enhanced the rate of exclusive breastfeeding among preterm infants.^[11,12,17]^ While prior studies have reported that FICare decreases the length of hospital stay in neonatal units – a result not observed in our study – this discrepancy may be attributed to a significant difference in the birth weights of preterm infants between the groups. Notably, FICare based on Swanson caring theory appeared to positively influence the growth and development of preterm infants up to at least 6 months corrected age. Repeated measures ANOVA revealed that this effect was most pronounced in terms of weight (*P* = .002) and length (*P* = .041), which were significantly improved compared to the control group, although no significant difference was observed in head circumference (*P* = .462). Furthermore, the linear regression analysis of growth and developmental indicators at each time point confirmed a positive association with FICare based on Swanson caring theory (Table 4). These results suggest that the model of care provided does indeed influence the developmental outcomes of preterm infants, highlighting the significance of Swanson caring theory and the FICare approach in neonatal care practices.

A critical consideration in interpreting our growth findings is the phenomenon of catch-up growth among preterm infants, due to the intervention group having a significantly lower baseline birth weight. However, the multivariable models suggest that the intervention remained a significant independent predictor of weight and length at corrected ages after this adjustment (Table 4). Furthermore, the concurrent improvements in head circumference and length, in conjunction with enhanced breastfeeding rates and reduced readmissions, provide evidence that extends beyond mere weight catch-up.

In addition, this study suggests that nursing care not only contributes to the developmental outcomes of premature infants but also helps alleviate parental anxiety and enhance their sense of parenting competence.^[18–20]^ The clinical importance of the observed outcomes was further interpreted according to established minimal clinically important difference criteria, using a 0.5 standard deviation threshold.^[21]^ For parental state and trait anxiety, a score change of over 5 points was regarded as the threshold for clinically meaningful improvement, consistent with established minimal clinically important difference criteria for the STAI scale in parental populations.^[22]^ The between-group difference in our study exceeded this cutoff (Table 2), indicating that the intervention not only differed statistically but also produced tangible clinical relief of parental anxiety. However, a notable decrease in S-AI scores was also observed among fathers in the control group, which may be attributed to the differing family roles assumed by mothers and fathers. For parenting sense of competence, a score increase of over 4 points was defined as clinically meaningful.^[23]^ The observed intergroup difference also reached this threshold, demonstrating that Swanson theory-guided FICare could substantially enhance parents’ actual caregiving confidence and ability.

A landmark multicenter, multinational cluster randomized controlled trial by O’Brien et al has demonstrated that FICare improved infant weight gain, decreased parent stress and anxiety, and increased high-frequency exclusive breastmilk feeding at discharge.^[24]^ Our study presented a structured FICare approach guided by Swanson caring nursing theory and extended the FICare intervention to the corrected gestational ages of 1, 3, and 6 months for preterm infants.

This study has several limitations. First, this was a single-center non-randomized cohort study, with group allocation based on parental willingness rather than randomization, which may introduce inherent selection bias. Meanwhile, the non-randomized observational design limits the ability to draw definitive causal inferences. Second, although repeated measures ANOVA and multivariate linear mixed model analysis were conducted, the baseline imbalance between the 2 groups may have introduced potential confounding interference. Third, complete blinding was not achieved. Although objective growth measurements were assessed by blinded staff, parents and nursing implementers could not be blinded to group assignment, which may lead to subjective response bias in parental self-reported anxiety and parenting competence questionnaires. Fourth, the current study cannot distinguish which specific component of Swanson theory-guided FICare program primarily contributed to the observed beneficial outcomes. Fifth, the implementation of this standardized FICare model relies on sufficient professional medical and nursing manpower, which may restrict its large-scale popularization and widespread clinical application.

## 5. Conclusion

This cohort study suggests that Swanson theory-guided FICare may be associated with improved growth outcomes, but causal inference is limited by the non-randomized design. Further larger-scale randomized controlled trials are warranted to verify the long-term effectiveness of this nursing model.

## Acknowledgments

We extend our sincere gratitude to all the nursing staff in the NICU for their dedicated support and professional care.

## Author contributions

**Conceptualization:** Huijie Zhu.

**Data curation:** Lili Chen.

**Formal analysis:** Lili Chen.

**Methodology:** Lili Chen.

**Supervision:** Huijie Zhu.

**Writing – original draft:** Lili Chen.

**Writing – review & editing:** Huijie Zhu.
